# Designing and implementing an electronic dashboard for disease outbreaks response - Case study of the 2013-2014 Somalia Polio outbreak response dashboard

**DOI:** 10.11604/pamj.supp.2017.27.3.11062

**Published:** 2017-06-22

**Authors:** Raoul Kamadjeu, Caroline Gathenji

**Affiliations:** 1 United Nations Children’s Fund Eastern and Southern Africa Regional Office (UNICEF ESARO), Nairobi, Kenya; 2 World Health Organization, Kenya Country Office, Nairobi, Kenya. All the authors were affiliated with the World Health Organization (Polio Eradication Program), Somalia Liaison Office when the project was implemented

**Keywords:** Dashboard, polio outbreak, Somalia, informatics, infectious disease surveillance, electronic surveillance

## Abstract

In April 2013, a case of wild polio virus (WPV) was detected in the Somalia capital Mogadishu. This inaugurated what is now referred to as the 2013-2014 Horn of Africa Polio outbreak with cases reported in Somalia, Kenya and Ethiopia. By the notification of the last polio case in August 2014, 223 cases of WPV had been reported in Somalia, Kenya and Ethiopia of which 199 in Somalia alone. The outbreak response required timely exchange of information between the outbreak response coordination unit (in Nairobi) and local staff located in multiple locations inside the country. The need to track and timely respond to information requests, to satisfy the information/data needs of polio partners and to track key outbreak response performance indicators dictated the need to urgently set up an online dashboard. The Somalia Polio Room dashboard provided a graphical display of the polio outbreak data to track progress and inform decision making. The system was designed using free and open sources components and seamlessly integrated existing polio surveillance data for real time monitoring of key outbreak response performance indicators. In this article, we describe the design and operation of an electronic dashboard for disease surveillance in an outbreak situation and used the lessons learned to propose key design considerations and functional requirements for online electronic dashboards for disease outbreak response.

## Introduction

Polio is a disease targeted for elimination since 1988 when the World Health Assembly voted to eradicated polio [1]. With an estimated 300,000 cases per day reported globally at the beginning of the Polio Eradication Initiative, the number of polio cases plummeted to less than 100 cases per year by the end of 2012. Despite these tremendous efforts, the persistence of polio transmission in endemic countries of Nigeria, Afghanistan and Pakistan and the resurgence of polio in non-endemic countries threatened the eradication effort and prompted the declaration of polio as a public health emergency of international concern by the World Health Organization (WHO) Director in May 2014 [2]. In April 2013, a case of Wild Polio Virus (WPV) was reported in Mogadishu the capital city of Somalia. Initially located within the capital, the polio outbreak quickly expanded to other districts of the country and in the neighboring countries of Kenya and Ethiopia. By the end of 2014, 223 polio cases were notified in three countries of the Horn of African (HoA) (Somalia, Ethiopia and Kenya) with 199 cases in Somalia alone. Immediately after notification of the first polio case in Somalia in April 2013, an outbreak response plan was set into motion in compliance with polio outbreak response guidelines. The ongoing insecurity and the ban of vaccination activities by various anti-government groups in the country required a multi-layered response structure; a national outbreak response coordination group based in Nairobi provided technical guidance and monitored the implementation of outbreak response activities by an army of field-based polio officers located in Somalia at community, district, regional and zonal levels. This multi-layer structure required timely information to monitor progress and impact of outbreak response activities.

## What is a dashboard?

In a management and information system standpoint, a dashboard is “an easy to read, often single page, real-time user interface, showing a graphical presentation of the current status (snapshot) and historical trends” [3]. Stephen Few beautifully described dashboards as “visual displays of the most important information needed to achieve one or more objectives, consolidated and arranged on a single screen so the information can be monitored at a glance” [4]. Dashboards therefore bear key important characteristics: they provide a rich visual interface that usually summarizes complex data streams into easy to read information; they are designed to specifically provide quick and easy-to-read snapshots of ongoing and sometimes complex processes.

### Rational for designing a dashboard in outbreak response

Polio outbreak response is data and information intensive. Timely and accurate information from field activities are required 1) to monitor key performance indicators to measure progress towards planned goals and timely identified deviations from planned goals and implement corrective measures; and 2) to assess the impact of outbreak response activities. Dashboards are not new in public health; however, their use in disease outbreak response at country level by national programs is not very well documented. Notable examples of the use of dashboards include multiscreen displays of disease and outbreak information at the Centers for Disease Control Emergency Operation Centers [5] or at the Strategic Health Operation Centers (SHOCK Room) of the World Health Organization Global Alert and response Network Center [6]. The Somalia Polio Room Dashboard is an online data visualisation platform providing an integrated and rich visual display of various components of the polio outbreak response and acute flaccid paralysis (AFP) surveillance activities. The system was designed to timely improve access to information to a wide and geographically scattered audience while reducing the need for routine daily analysis and ad-hoc information requests.

## Architecture

**Figure 1:**
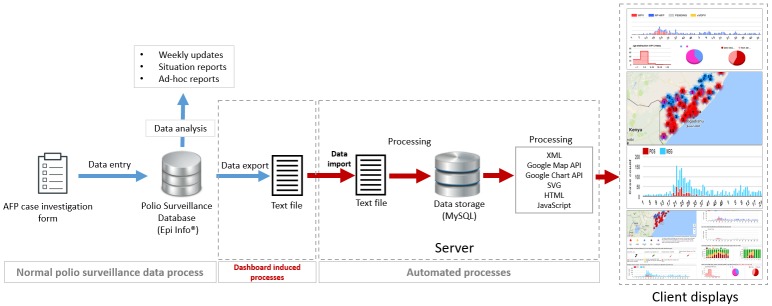
Somalia Polio Room Dashboard system architecture

Figure 1 provides a description of the overall architecture of the system. The online system was designed using open source and freely accessible tools. Programming was done using the open source web-scripting language PHP [7] and was running on an APACHE web-server. MySQL database management system served as the primary data storage engine. Maps were generated using Google Maps [8] and Scalable Vector Graphic (SVG) technology; graphs were generated using Google Charts tools [9]. An administration password protected area allowed for basic configuration and data uploads. A visualization area, specifically designed for high resolution large monitors gave access to a rich visual environment combining maps, tables and graphs.

**Data inputs:** the system was designed to use acute flaccid paralysis (AFP) data as the primary data source. AFP describes a sudden onset of weakness or paralysis, affecting mostly motor muscles of lower limbs in children. AFP is the main clinical presentation of poliomyelitis disease [10]. Patients, mostly children under 15-years of age, presenting with AFP are the primary target of polio surveillance. AFP cases are reported through the polio surveillance network consisting of health facilities and community informants. Suspected case of AFP are investigated by polio surveillance officers using standardized AFP investigation forms. Two stool samples are collected for each AFP cases and sent to an accredited laboratory to confirm or rule out polio. Data elements of each individual AFP cases are recorded in the investigation form and entered into a computerized national database known as the AFP national database.

The AFP database served as the primary source of data for the dashboard to avoid interfering with ongoing AFP surveillance and data entry processes; no new data source was created or required. A tab-separated text file containing selected AFP data elements (epidemiological number – epidemiologic Number, date of birth, sex, date of onset of paralysis, district of onset of paralysis, number of doses of oral polio vaccine received, final classification) was generated from the AFP main database and imported into the system. On upload, the text file was processed and the key data elements stored in a table in the MySQL database. To accommodate for early notification of cases, a user protected area allowed for the manual entry of AFP cases by surveillance officers located in districts through a customized online form. Additional data streams used by the system are shown on Figure 1.

**Geo-mapping:** the systems integrates Google Map API to display the location of AFP cases. Since geolocation information of AFP cases are not available, we used the latitude and longitude coordinates of the capital city of district of residence of the AFP case as a proxy to the geolocation of the AFP case. The altitude and longitude data of all district city capitals were extracted from the geo-name server table [11] for Somalia were stored as a table in the system database. The geolocation table was secondarily linked to the AFP table to generate an XML file used to display the geo-location of AFP cases on Google Map using the Google Map API.

**Figure 2:**
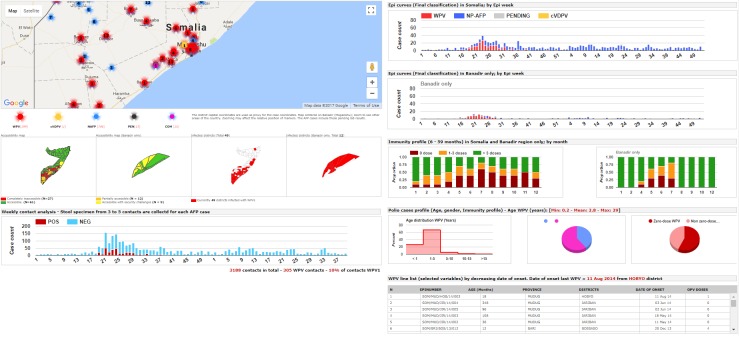
sample display 1 of the Somalia Polio Room Dashboard combining graphs, maps

**Figure 3:**
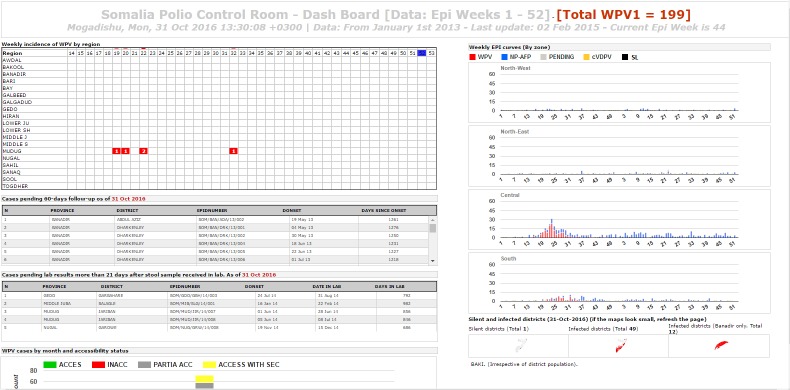
sample display 2 of the Somalia Polio Room Dashboard combining graphs, maps and a table summarizing key variable for all polio cases

**Figure 4:**
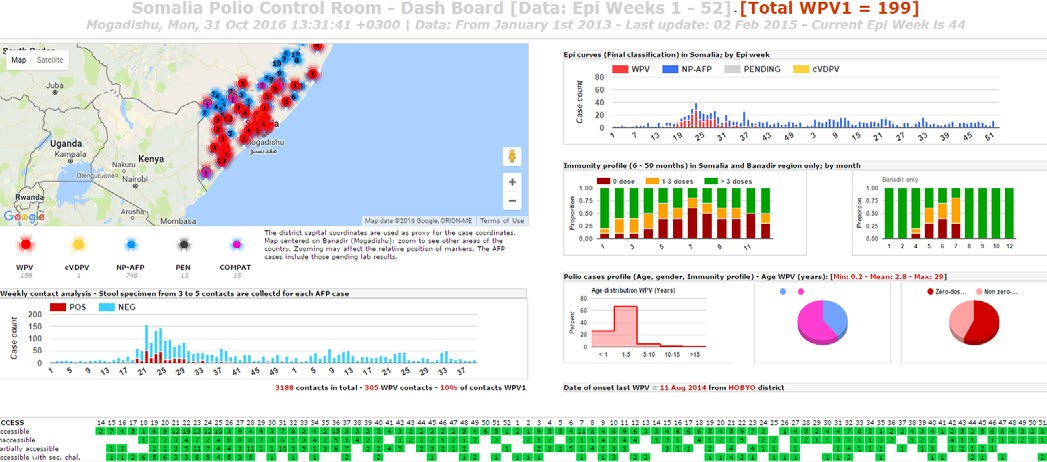
sample display 3 of the Somalia Polio Room Dashboard showing critical performance indicators for Acute Flaccid Paralysis surveillance

**Graphical display:** the visual interface of the system was specifically designed for display on high resolution/large monitors in the outbreak control room located in the WHO Somalia Liaison Office in Nairobi. Six rich individual displays existed and combined maps, graphs and tables summarizing the current status of outbreak response and AFP surveillance activities. Figure 2, Figure 3 and Figure 4 provide examples of such displays. A password protected areas allowed access to additional display and system customization features. The use of open source and free components coupled with the availability of a rich polio dataset allowed for a rapid customization of the system display to fit ongoing program monitoring needs.

## Outcome

During its 15 months of existence (July 2013 to Oct 2014), the Somalia Polio Room Dashboard, by integrating various outbreak and surveillance response data sources into rich graphical interfaces provided an easy-to-read information to polio outbreak response managers, responders and immunization partners located in various areas inside and outside of the organization. A polio outbreak response involves a multitude of players at various levels of the same organization or outside of the organization. The ongoing analysis and sharing of information with all these players can place an undue burden on surveillance staff and data managers. With the Somalia Polio Room Dashboard, we were able to reduce the need of daily analysis and sharing of outbreak information, thereby release surveillance staff from the need to response to multiple information requests from various sources.

The methodological innovation of the system can be viewed at various levels: 1) we demonstrated the possibility of designing, at country level, an effective data visualisation tool using available and free technology in a limited amount of time; 2) we demonstrated that the use of a visualisation tool accessible to all partners can significantly reduce the time spent on routine analysis and other reports.

**Lessons learned on key considerations for dashboards for outbreak response** In his 2006 essay on dashboard design, Stephen Few provided 13 pitfalls for dashboard design [4]. These pitfalls remain relevant and should be kept in mind in the design of successful dashboards for infectious disease outbreaks. Through the design and operation of the Somalia Polio Dashboard, we identified additional important pre-requisite that should guide the design of dashboards for disease surveillance. Before setting up a dashboard in an outbreak response context, we found it important to ensure that (Table 1):

**Table 1:** summary of key issues to consider for electronic dashboards for disease outbreak response

Issues to consider	Comments
Establish the need of the dashboard	Are existing data visualization, analysis and sharing mechanisms and tools sufficient or is there a clear need for additional or expanded data visualization tools?
	Is there an audience for the dashboard?
Establish that the dashboard will provide critical advantage over existing data exchange mechanisms and formats	Will the dashboard will provide an advantage over what exists now?
Establish that the dashboard will be needed for an acceptable period of time	For how long will the dashboard be needed
Establish that a structured and regularly updated data streams exist	The visual display of the dashboard should be regularly dashboard
	The dashboard should use existing data and should not require new data collection processes.
The workload required to update and operate the dashboard should be nil to minimal on the outbreak response staff	During an outbreak, the surveillance staff including data managers and data entry clerks are overburdened and should not be distracted by new processes or long trainings on how to operate the dashboard
The dashboard should be design for scalability and flexibility	This is necessary to accommodate the changing information requirements of the outbreak response; it should be easy to add, remove or modify the visual display
Use of available, preferably open sources or free technology and web-services	To minimize the cost of the dashboard development.

1. **The need for a dashboard is established:** are existing data visualization, analysis and sharing mechanisms and tools sufficient or is there a clear need for additional or expanded data visualization tools? Knowing who has interest in accessing the dashboard should be the main determinant of whether or not to pursue the project of developing a dashboard
2. The dashboard should provide **critical advantages** over existing data exchange mechanisms and formats
3. **The need of the dashboard should be established for a reasonable period of time:** establishing that the need for a dashboard will be sustained for an acceptable period of time should help decide whether investing time and efforts into the development of a dashboard is warranted
4. **Structured and regularly updated data streams exist:** to feed the need to constantly update the rich visual display of the dashboard; a dashboard that is not updated regularly loses its appeal. The dashboard should use existing data and should not require new data collection processes
5. **The workload and skills required to update and operate the dashboard should be nil to minimal on the outbreak response staff:** during an outbreak, the surveillance staff including data managers and data entry clerks are overburdened and should not be distracted by new processes or long trainings on how to operate the dashboard
6. **The dashboard should be designed for scalability and flexibility:** and should easily accommodate the changing information requirements of the outbreak response; it should be easy to add, remove or modify the visual display or other processes
7. Use of available, preferably **open sources or free technology** and web-services to minimize the cost of the dashboard development

## Conclusion

We have demonstrated that it is possible to develop an online electronic dashboard for outbreak response using existing open source and free tools. The development of such dashboard requires technical expertise but should also be carefully examined in the light of the considerations we identified. The Somalia Polio Room Dashboard clearly demonstrates the usefulness of electronic dashboard in outbreak response through their ability to improve information sharing and minimize the time spent on multiple information requests; their use should therefore be encouraged. The technical expertise in computer programming, data management and software design required to set-up functional and useful electronic dashboards remains a limitation. To address these limitations, technical partners including WHO should develop and avail ready-to-use/easy-to-setup dashboards for outbreak response.

## Competing interests

The authors declare no competing interests.

## References

[1] Polio Eradication Initiative

[2] WHO WHO statement on the meeting of the International Health Regulations Emergency Committee concerning the international spread of wild poliovirus..

[3] Wikipedia (2016). Dashboard (business)..

[4] Few S Common pitfalls in Dashboard Design..

[5] CDC CDC Emergency Operations Center PHPR..

[6] WHO Strategic Health Operations Centre (SHOC)..

[7] PHP Hypertext Preprocessor..

[8] Google Google Maps APIs for Web..

[9] Google Charts by Google..

[10] WHO WHO-recommended surveillance standard of poliomyelitis..

[11] NGA GNS Home..

